# Unsupervised bivariate data clustering for damage assessment of carbon fiber composite laminates

**DOI:** 10.1371/journal.pone.0242022

**Published:** 2020-11-13

**Authors:** Zazilah May, M. K. Alam, Muhammad Shazwan Mahmud, Noor A’in A. Rahman

**Affiliations:** 1 Electrical and Electronic Engineering Department, Universiti Teknologi PETRONAS, Seri Iskandar, Perak, Malaysia; 2 Mechanical Engineering Department, Universiti Teknologi PETRONAS, Seri Iskandar, Perak Darul Ridzuan, Malaysia; University of Vigo, SPAIN

## Abstract

Damage assessment is a key element in structural health monitoring of various industrial applications to understand well and predict the response of the material. The big uncertainty in carbon fiber composite materials response is because of variability in the initiation and propagation of damage. Developing advanced tools to design with composite materials, methods for characterizing several damage modes during operation are required. While there is a significant amount of work on the analysis of acoustic emission (AE) from different composite materials and many loading cases, this research focuses on applying an unsupervised clustering method for separating AE data into several groups with distinct evolution. In this paper, we develop an adaptive sampling and unsupervised bivariate data clustering techniques to characterize the several damage initiations of a composite structure in different lay-ups. An adaptive sampling technique pre-processes the AE features and eliminates redundant AE data samples. The reduction of unnecessary AE data depends on the requirements of the proposed bivariate data clustering technique. The bivariate data clustering technique groups the AE data (dependent variable) with respect to the mechanical data (independent variable) to assess the damage of the composite structure. Tensile experiments on carbon fiber reinforced composite laminates (CFRP) in different orientations are carried out to collect mechanical and AE data and demonstrate the damage modes. Based on the mechanical stress-strain data, the results show the dominant damage regions in different lay-ups of specimens and the definition of the different states of damage. In addition, the states of the damage are observed using Scanning Electron Microscope (SEM) analysis. Based on the AE data, the results show that the strong linear correlation between AE and mechanical energy, and the classification of various modes of damage in all lay-ups of specimens forming clusters of AE energy with respect to the mechanical energy. Furthermore, the validation of the cluster-based characterization and improvement of the sensitivity of the damage modes classification are observed by the combined knowledge of AE and mechanical energy and time-frequency spectrum analysis.

## Introduction

Recently, potential applications of carbon fiber composite materials have been explored because of the material’s properties, which include high stiffness and shape stability, as well as being lightweight, non-conductive, and economic. Carbon fiber composite materials have been increasingly used in many applications, including those connected with the aerospace and space industries, fuel cell cars, sporting goods, turbine blades, and automotive parts. These composite materials attract more attention than traditional materials such as concrete, wood, and metal. However, despite having many great advantages in many applications, they are susceptible to impact damage due to the lack of reinforcement in the out-of-plane direction [1]. In addition, fiber breakage and matrix cracking induced by the de-lamination process are becoming common failure types when dealing with composite materials. Thus, damage or defect assessment is necessary for the early detection of potential failure.

Defect assessment is a key element in quality inspection in various industrial applications. The quality of composite materials and the profitability of the work depend greatly on the manufacturing process. The presence of inclusions and flaws during the manufacturing process causes geometrical discontinuities, affecting failure strength and the overall material performance [2]. The AE technique offers promising results in the field of structural health monitoring (SHM) to detect the modes of damage to composite materials. AE sources can originate matrix cracking, carbon/fiber de-bonding, fiber breakage, and delamination damage in composite laminates.

There are several studies that provide quantitative results on structural health assessment using AE. Besides composite structure, the AE technique has proven successful in the real-time monitoring of damage growth in structural components such as granite [3], metal, and alloys [4], concrete [5], foam [6], and polymeric coating [7], by correlating the parameters of AE data to the damage mechanism. In real applications, damage evaluation greatly depends on experimental results rather than numerical simulations because of the multiple damage mechanism, which can cause significant reductions in both tensile and compressive strength [8]. Current practice shows that AE data are able to provide different parameters to identify the mode of failure occurring in composite fiber materials. AE data can be analyzed in terms of the evolvement of the amplitude, counting, and energy. Kaltermidou et al. [9] investigated the damage progression in the composite structure using the rise time of the AE data. R Khamedi et al. in [10] identified the failure mechanisms of unidirectional composites using wavelet packet transform before processing it with the absolute energy of the AE data. Munoz et al. in [11] combined infrared thermography and AE data amplitude techniques to identify the damage evolution in composite fiber. Furthermore, several studies on the AE data generated from mechanical testing have been reported. Various researchers studied the correlation of AE features on composite specimen deformation by tensile testing [9–13]. The most of researchers highlight the investigation of damage progression in the composite is still a challenging task, especially when multiaxial stress state occurs.

In addition, AE data collected from tensile and bending tests were grouped and analyzed to understand the behavior of composite materials under different forces [14]. The mode of damage that occurs during tensile testing is successfully identified by AE. Effective and efficient analysis is still a relevant topic of study because the data are unique. A trend of embedding a piezoelectric sensor in the sample structure is quite popular among researchers when dealing with AE. Sahir et al. [15] monitored composite materials by embedding piezoelectric sensors in the structure subjected to mechanical tests. Even though the embedded sensor showed higher sensitivity compared to a sensor mounted on the surface of the structure, this technique is more costly and has limited applicability, as the sensor has to be integrated during the production process. Hence, the AE technique in structural health monitoring is able to provide sufficient features to identify and predict composite failure modes, including matrix cracking, debonding, fiber fracture, and delamination. The AE technique employs to monitor the acoustic response of the composite laminates throughout the tensile tests. AE is a non-destructive testing (NDT) method which has been used in several SHM applications for the damage assessment of carbon fiber composite materials [14, 16–18] and other engineering materials [15] either as a self-standing method or in integration with other NDT techniques [9]. However, there is no strong correlation of the AE activity to multiaxial stresses introduced in composite materials found in the literature. In this work, we investigate the association between the AE features and mechanical data for identifying damage and failure initiations of CFRP specimens. Afterward, we conduct multiple tensile tests on CFRP specimens in two different orientations to collect mechanical and AE data. Furthermore, we classify the initiation and progress of the modes of damage and failure in the tested specimens using mechanical stress-strain data. Besides, the SEM analysis is carried out on tested specimens to verify the modes of damage. In addition, an adaptive sampling technique is introduced to resample the AE data by eliminating redundant data samples to fit the AE data to the bivariate clustering model. Afterward, the correlation between AE and mechanical data is analyzed. Moreover, an unsupervised bivariate data clustering technique is utilized to classify the states of damage of CFRP specimens based on the combined knowledge of AE and mechanical data. Finally, sentry function analysis is performed to verify and improve the sensitivity of various modes of damage initiations and failure classification by the combination of AE and mechanical data.

## Materials and tests

This section explains the various features and characteristics of the given specimens. The details about the experimental tests for mechanical and AE data acquisition are discussed.

### Material specimens

Specimens were composed using XPREG^®^XC110 Carbon Fiber Epoxy Prepeg (0.25 mm thickness) supplied by Easy Composites Ltd. Staffordshire, United Kingdom. XPREG^®^XC110 Carbon Fiber Epoxy Prepreg is made from a uniform plain weave of Pyrofil TR30S high strength 3 k carbon. The specimen was prepared using the single vacuum bagging method and was subjected to various temperatures up to 120°C and a constant pressure of 1 MPa using XC110 epoxy adhesive to produce a (320 x 250 x 2.5) mm plate geometry. The final laminate consisted of ten layers of uniform plain weave with constant weave dimensions. The specimens were cut into two types of lay-up which were 45° and 90° lay-ups with final dimensions of (270 x 250 x 2.5) mm using a diamond-coated abrasive cutter. The 45° and 90° layouts were determined by referring to the initial 0° of the vertical direction of the plain weave. The specimen underwent pre-conditioning at 23°C for 16 hours in a desiccator to stabilize the humidity prior to the tensile test.

### Tensile test

The mechanical testing was performed using a tensile test following the ASTM D3039/D, a standard testing method to generate damage modes of composite material. Both types of specimens were fixed between the jaws of the machine to undergo testing. These tests were conducted using a universal tensile machine (UTM) manufactured by Gotech Testing Machine Inc. (AI-7000L) with a 15 kN capacity, at room temperature. For each type of lay-up, at least four specimens were tested. The average value and the standard deviation were then recorded.

### Acoustic emission data acquisition

**Fig 1:**
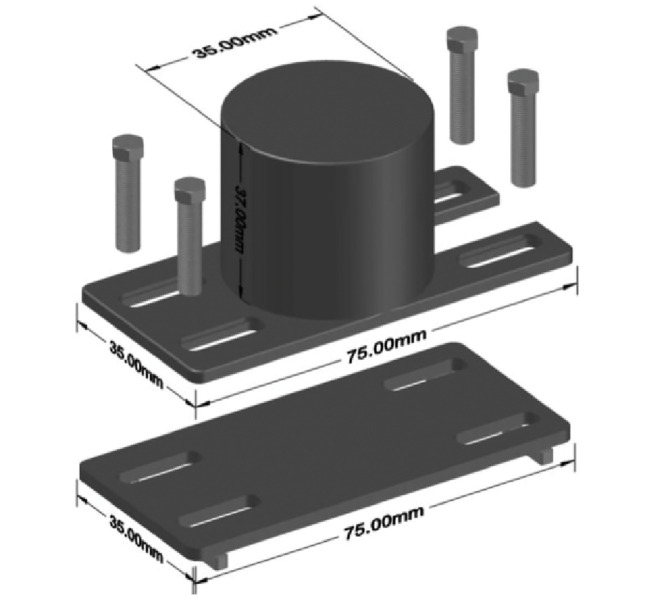
AE sensor holder drawing.

The AE sensor was installed onto the specimen to monitor the damage of the composite material continuously during the tensile tests. However, installing the AE sensor is complicated when dealing with a composite specimen. Unlike metallic specimens, conventional magnetic sensor holders are not applicable to composite specimens. There is a risk of breakage when the specimen undergoes the harsh operating test. Thus, the AE sensor must be mounted onto the specimen properly in such a way that the AE data are efficiently transmitted. Fig 1 shows the sensor holder design, which is mainly used for composite specimens. The holder was made from lightweight aluminum, making it easy to handle. The sensor was placed in the middle of the holder.

**Fig 2:**
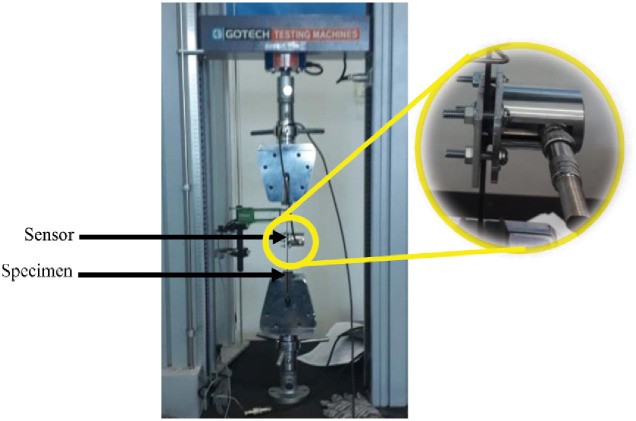
The acoustic emission (AE) sensor position on the specimen, focusing on the location of the sensor and the special sensor holder.

**Fig 3:**
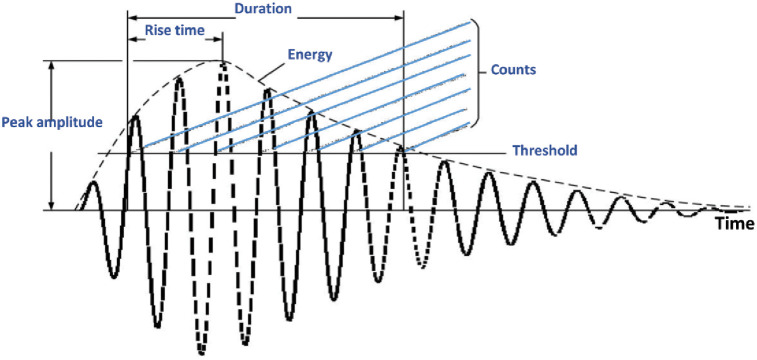
Features of an AE waveform.

AE data and mechanical data were recorded simultaneously when the tensile tests started and continuously acquired the data during the tests. AE Data were monitored on one channel of the data acquisition system with a built-in low-noise preamplifier. In this work, a piezoelectric sensor was used for data acquisition and R6I-Auto Sensor Test (AST) was executed to fix the threshold to 40 dB. The sensors were attached to the specimen together with the coupling agent and secured with our modified sensor holder to hold the sensor onto the specimen in Fig 2. The whole system including the sensor was supplied by Physical Acoustics Corporation (USA). The pencil lead break procedure was used to calibrate the acquisition system prior to tensile testing. Values for peak definition time (PDT), hit definition time (HDT), and hit lockout time (HLT) was employed in the acquisition software. The values were PDT = 400 *μ*s, HDT = 800 *μ*s, and HLT = 1000 *ms*. These values depend on the type and nature of the material. The main AE data parameters measured throughout the tensile test are depicted in an example of the AE waveform in Fig 3. In this work, the classification of AE parameters was done using input features namely rise time, counts, amplitude, duration, data strength, root mean square (RMS), absolute energy, and peak frequency. A short description of each feature in the waveform is as follows:

- **Rise time**—the time from first threshold crossing to the highest voltage point on the waveform
- **Counts**—number of times that data cross the detection threshold
- **Amplitude**—highest voltage in the AE waveform, expressed on the dB AE amplitude scale
- **Duration**—the time from first to last threshold crossing (*μ*s)
- **Signal strength**—time integral of the absolute signal voltage expressed in pVs (picovolt-seconds) referend to the sensor before any amplification.
- **Root mean square (RMS)**—voltage during a period based on software programable time constant, referend to the input to the signal processing board
- **Absolute energy (Abs. energy)**—time integral of the square of the signal voltage at sensor before any amplification divided by a 10kΩ impedance and express in aJ (attojoule)

### Morphology of specimen

The morphologies of the specimens were taken using a scanning electron microscope (SEM) manufactured by ZEISS (Zeiss Supra 55 VP). A small breaking part of the specimen was cut and held over a holder and sprayed with gold (Au) to enable and improve the quality of the image taken prior to SEM analysis.

## Proposed partitioning-based adaptive data sampling and clustering technique

Partitioning-based data clustering is a well-known unsupervised machine learning technique that is broadly used in the data mining field to discretize the time-series data into classes. Time-series data refer to the systematic data collection process where data are sampled by the sensors at every constant time interval. Partitioning clustering technique groups similar data into clusters to reduce the data redundancy and characterize the classes of data. This paper introduces a new partitioning-based data clustering technique which is mainly divided into two modules. The first module is histogram-based adaptive data sampling which partitions similar data into the pre-defined number of histogram bins. Afterward, we choose the number of desired data samples from each bin adaptively by eliminating data redundancy. The concept of histogram-based adaptive data sampling is adopted from the work in [19]. The second module is an unsupervised clustering technique named bivariate K-Means clustering which groups bivariate data into clusters to classify the relationship among different groups of data of a particular dependent variable with respect to the groups of data of another independent variable. The development steps of the proposed technique are presented in **Algorithm 1** where the procedural steps according to (*lines* 1–16) sampling data adaptively and based on (*lines* 17–27) clustering bivariate datasets into different groups.

### Adaptive data sampling

An adaptive data sampling is utilized in this paper to eliminate the desired number of AE redundant data samples before the bivariate data clustering technique is performed. In order to maintain equality in the number of data samples of the different datasets during bivariate data clustering, the proposed adaptive sampling technique is utilized. The development steps of this module are as follows:

Initially, the given dataset *X*_*i*_ where *i* = 1, 2, 3, ·····, *n* is used as input original data samples. Then, the desired number of bins *N*_*b*_ to partition data into different groups is defined. The range Δ_*d*_ of the given dataset *X*_*i*_ is computed to determine the constant discretization step *W*_*d*_ using the Equation in 1 and 2.

$$\begin{array}{c} \Delta_{d}=\text{max}\;X_{i}-\text{min}\;X_{i}, \end{array}$$ (1)

$$\begin{array}{c} W_{d}=(\frac{\text{max}\;X_{i}-\text{min}\;X_{i}}{N_{b}}). \end{array}$$ (2)

**Algorithm 1**: Adaptive Data Sampling and Unsupervised Clustering Algorithm

**Input**: *X*_*i*_—Original data samples

**parameter**: $N_{b}=\left|X_{i}^{{}^{\prime }}\right|$—Number of bins

**Output**: *c*_1_, *c*_2_, *c*_3_, ⋯*c*_*k*_—Number of clusters

1 Compute the range Δ_*d*_ of *X*_*i*_ and determine the discretization step *W*_*d*_

2 Update all bin edges *E*_*k*_ and set the limit of constant bin intervals recursively

3 **for** *i* ← 0, 1, 2, 3, ·····, *X*_*i*−1_ **do**

4  Compute *Histogram* based on the limits of each bin interval

5  Discretize the data of *X*_*i*_ into different bins *b*_*k*_ based on the given intervals

6  *N*_*e*_ = 0 {Number of elements in each *b*_*k*_};

7 **end**

8 **for** *k = 0 to b*_*k*−1_ **do**

9  Compute *N*_*e*_ of each *b*_*k*_;

10  Compute the desired elements $N_{k}=\lceil \frac{N_{e}\times |X_{i}^{{}^{\prime }}|}{\left|X_{i}\right|}\rceil$ from each *b_k_*;

11  Standardized the number of elements $Sb_{k}(l)=\|\frac{b_{k}(l)-\mu_{k}}{\sigma_{k}}\|$ of each *b_k_*;

12  Select the lowest standardized elements *Sb*_*L*_ where |*Sb*_*L*_| = *N*_*k*_;

13  De-standardized the *Sb*_*L*_ by *Db*_*k*_ = (*Sb*_*k*_(*l*) × *σ*_*k*_ + *μ*_*k*_);

14  Concatenate samples $X_{i}^{{}^{\prime }}=X_{i}^{{}^{\prime }}+Db_{k}\;\{\text{where}\;\text{initial}\;X_{i}^{{}^{\prime }}=\left[0\right]\}$;

15 **end**

16 Aggregated samples $X_{i}^{{}^{\prime }}$, where $X_{i}^{{}^{\prime }}<X_{i}$;

17 Initilize the cluster centroids randomly *K* = *μ*_1_, *μ*_2_, *μ*_3_, ⋯*μ*_*k*_{where *k* = 1, 2, 3, ⋯*K*};

18 **repeat**

19  **for** *i = 1 to* $X_{i}^{{}^{\prime }}$ **do**

20   Assign $X_{i}^{{}^{\prime }}$ to the nearest *μ_k_*;

21   $\ell =\sum_{k=1}^{K}\sum_{i=1}^{n}{\left\|X_{i}^{{}^{\prime }}-\mu_{K}\right\|}^{2}=\sum_{k=1}^{K}\sum_{i=1}^{n}\gamma_{ik}{\left\|X_{i}^{{}^{\prime }}-\mu_{k}\right\|}^{2}\{\text{where}\;\gamma_{ik}=\{{}_{0\;\text{else}}^{1\;\text{if}\;\text{k}=agr_{l}^{\text{min}}{\left\|X_{i}^{{}^{\prime }}-\mu_{k}\right\|}^{2}}\}$;

22  **end**

23  **for** *k = 1, 2, 3, ⋯K* **do**

24   $c_{k}=\sum_{i=1}^{n}\gamma_{ik}$ {Number of elements assigned to cluster k};

25   $\mu_{k}=\frac{1}{c_{k}}\sum_{i=1}^{n}\gamma_{ik}X_{i}^{{}^{\prime }}$ {*μ_k_* is set by averaging all assigned elements to k};

26  **end**

27 **until** *Convergence*;

The sequence of edges *E*_*k*_ of the bins is computed based on the arithmetic or linear sequence where *k* = 1, 2, 3, ·····, *K*. In a sequence of calculated edges, each new term is accounted by adding a constant interval-width *W*_*d*_ to the previous term. For example, the difference between any two adjacent edges is *W*_*d*_. The recursive definition is therefore as in Eq 3:

$$\begin{array}{c} E_{k}=E_{k-1}+W_{d},\;E_{1}=E_{0}+W_{d}, \end{array}$$ (3)

where the term *E*_*k*_ = *E*_1_, *E*_2_, *E*_3_, ⋯*E*_*k*_ and *E*_0_ = min(*X*_*i*_) is defined as the lower bound of edges as well as the bins *K* constant intervals (*E*_0_, *E*_1_], (*E*_1_, *E*_2_], (*E*_2_, *E*_3_], ⋯, (*E*_*k*−1_, *E*_*k*_]. The elements which fall between the lower-bound edge and upper-bound edge (e.g., *E*_0_ and *E*_1_) of a bin, then they are considered for that particular bin. Afterwards, we compute the number of elements *N*_*e*_ of each bin *b*_*k*_ to determine the number of desired elements *N*_*k*_ from each *b*_*k*_. The computed desired elements *N*_*k*_ are sampled from the elements of *b*_*k*_ those are the closest to the central value of that particular bin. The desired central elements of each *b*_*k*_ are chosen based on the standardized scores of the elements of each *b*_*k*_. Then, we de-standardize the selected elements from each *b*_*k*_ and aggregate them in $X_{i}^{{}^{\prime }}$.

### Bivariate data clustering

Data clustering is performed on the aggregated dataset $X_{i}^{{}^{\prime }}$ to group similar data into clusters for classification. The partitioning-based bivariate K-Means clustering algorithm is adopted in this paper which is able to group a particular dataset into different clusters with respect to the other various type of datasets. Moreover, this algorithm classifies the relation among different clusters of a particular dataset with the different clusters of other datasets. Initially, we determine the number of cluster centroids randomly. Then, the data samples of the given dataset $X_{i}^{{}^{\prime }}$ are assigned to the nearest centroid based on the euclidean distance method. Afterward, the number of assigned elements of each cluster is computed to determine the central value of that particular cluster. If the initial cluster centroids change after averaging, then repeat the clustering processes according to the **Algorithm 1** (*lines* 18–27) until the computed centroids are unchanged.

## Results and discussion

### Damage and failure regions characterization of carbon fiber composite based on mechanical data

**Fig 4:**
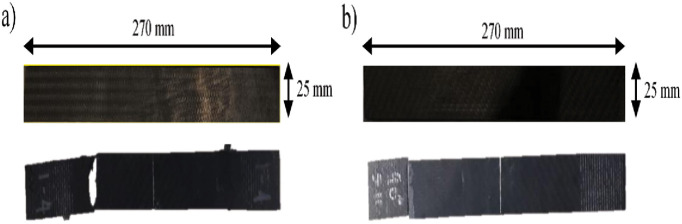
The images for specimens (a) 45° and (b) 90° lay-up before and after the tensile test.

**Fig 5:**
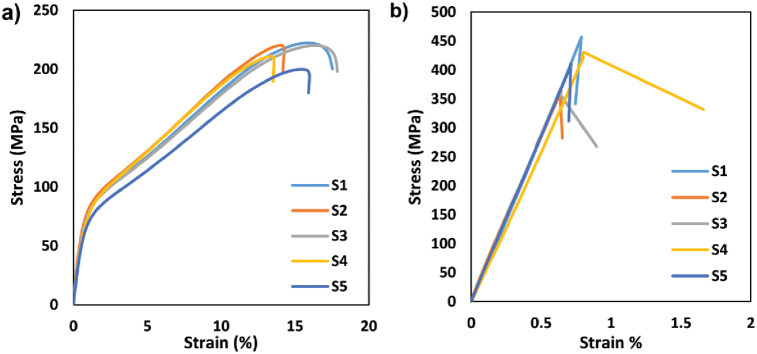
Graph stress-strain curve of specimen lay-ups (a) 45° and (b) 90°.

In this section, the mechanical data are utilized to identify the modes of damage to CFRP specimens. The data were collected from several tensile tests on similar CFRP specimens in two different orientations (45° and 90°). The total average duration of tensile test is 1200 *sec* (sampling rate 240 *ms*) for 45° lay-up and 250 *sec* (sampling rate 50 *ms*) for 90° lay-up respectively. The tensile strength of a CFRP test specimen is mainly conferred by the interfacial bonding between the carbon fibers and the epoxy matrix. Images of the post-mortem specimen for both lay-ups are shown in Fig 4. The type of failure mode is difficult to identify from visual observation. Commonly, a stress-strain relationship curve, as shown in Fig 5, is used to determine the mode of failure during the test. In general, all specimens showed a load increasing in the experimental curves due to the fiber pull-out within the epoxy matrix at the end of the test. However, the fiber orientation of the specimen definitely affected the mechanical properties including tensile strength and elongation.

A material can be classified into two types: a ductile material or a brittle material. As shown in Fig 5a, the 45° lay-up specimen is prone to ductile behaviour; the specimen tends to elongate more and the specimen breaks with a brushlike failure. Meanwhile in Fig 5b, the 90° specimen has a transient elongation and the sample breaks apart in an almost perfect transverse direction, showing brittle behaviour. This can be seen by referring to the comparison of the ultimate tensile strength (UTS) and strain percentages of the CFRP specimen, shown in Table 1. The second column of Table 1 indicates that the UTS value for the 90° specimen is approximately twice the value for the 45° specimen.

**Table 1:** Average ultimate tensile strength (UTS) and tensile strain for the 45° and 90° specimens.

Specimen lay up	Ultimate Tensile strength (MPa)	Tensile strain (%)
45°	214.70	15.03
90°	403.00	0.72

**Fig 6:**
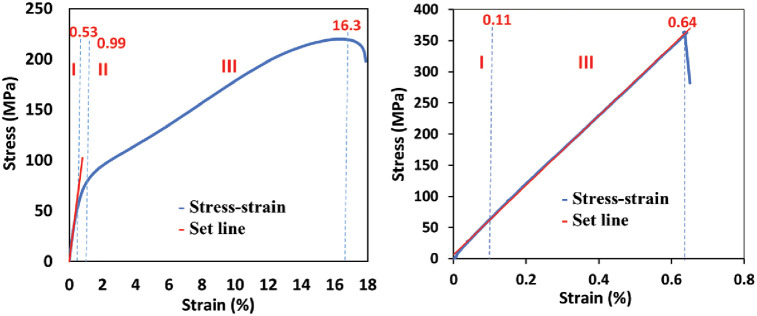
Designated regions for two different specimens. (a) 45° lay-up and (b) 90° lay-up.

For the 45° lay-up specimen, as shown in Fig 6a, the stress-strain curves were divided into three possible regions: Regions I-elastic region; Region II-yielding region; and Region III-strain hardening region. Region I is categorized between the initial point of strain and the proportional limit point. The slope of this region will generate the value of Young Modulus (Modulus of Elasticity) for the material. Region II is defined between the proportional limit point and the Yield Strength (YS). The YS point is computed from the value of 2% strain offset. While the Region III is characterized between the YS point and the Ultimate Tensile Strength (UTS) point.

In Region I, the material experience elastic behavior where it can restore its original shape after the load is removed. Beyond Region I, the elastic behavior is taking place by inelastic behavior so-called plastic deformation. In this phase, the molecular structure of the material starts to stretch until to the point of maximum stress that can be exerted before the material begins to permanently deform. This phenomenon is related to dislocation motion. At the exact point of where Region II starts, the material reaches its yield strength, and the necking effect takes place. The stress was continuously applied until the maximum stress point was reached, before the material bonding severely damage. The maximum stress point is known as the ultimate tensile strength (UTS).

A different phenomenon occurred on the 90° lay-up specimen in Fig 6b. The specimen experience only two regions are characterized such as Region I and Region III. The yielding region (Region II) is difficult to determine due to the specimen stretch almost directly proportional to the stress. The Region I and Region II overlap with the tangent line of the whole set line, making it impossible to distinguish the second region for this specimen. The stress-strain relationship of the specimen is almost linear indicated the specimen is brittle where the specimen will suddenly break after passing the UTS at the end of Region III. Thus, there were only two main regions for the 90° lay-up specimen.

### Scanning Electron Microscope (SEM) analysis

As predicted, the SEM images show the morphology of the failure mode after the specimens underwent the tensile test. The micro-structure of each specimen, with varying fiber orientation, impacted the mode of failure during the tensile test. In the following images, the mode of failure is noted. Similar trends of non-localized damage accumulation throughout the material are shown for the 45° and 90° lay-ups.

**Fig 7:**
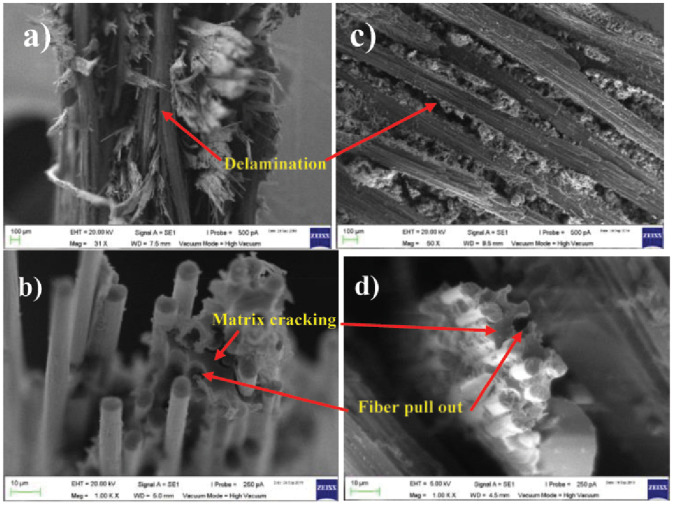
SEM morphology of each specimen: (a,b) represent the 45° specimen and (c,d) present the 90° specimen.

It is noted that the CFRP specimens are susceptible to impact damage due to the energy absorption during the tensile testing [8]. The energy is generally dispersed from the different combinations of composite damage, including matrix damage, fiber fracture, and fiber debonding, as illustrated in Fig 7.

The characteristics of surface fracture for the weak and strong interfaces are shown in Fig 7a. The fairly smooth surface, indicating a strong interface, can be seen on the 45° specimen, while the brushlike failure with an extensive pull-out effect indicates the extensive debonding which occurred in the 90° specimen. The difference in damage appearance of those two images emphasizes the importance of inter-facial strength based on the fiber layout orientation in controlling fracture behavior and toughness. This is due to the amount of absorbed energy from the fiber pull-out effect of the matrix. The delamination damage, as shown in Fig 7a and 7c, propagates from the transverse ply crack. Delamination occurs in Region III before the sample completely breaks. The break is induced by interlaminar shear stress which is escalated by matrix cracks or the ply stiffness mismatch.

Matrix damage and fiber fracture can be observed by the appearance of matrix cracking and fiber pull-out in both specimens in Fig 7b and 7d. For the 90° specimen, the longitudinal split through matrix fracture and debonding from the fiber is seen which is caused by the tensile load in the perpendicular direction of the loading axis. Meanwhile, matrix cracking and fiber debonding of both specimens are clearly noticed with the appearance of fiber pull-out from the matrix bond and fiber fracture.

### Damage and failure regions characterization of carbon fiber composite based on AE data clustering

In this section, the AE data are utilized to classify damage and failure regions of carbon fiber composite that are already characterized by the mechanical data. Five tensile tests were carried out on a similar type of specimen in two different orientations 45° and 90° for AE data collection. AE data were collected as a waveform in every 500 *ms* during tensile tests using a single AE sensor node. The average duration of AE data collection is equal to the duration of stress-strain data collection as mentioned in Section 27. Each waveform consists of 1024 data samples (sampling rate 488 *μ*s) and different AE features as highlighted in Fig 3. We consider average absolute energy (Abs energy) in this work which was mostly utilized in the literature [20] to characterize damage and failure phases of various types of composite materials. The average Abs energy was computed from each waveform energy feature. Afterward, we cumulate the average Abs energy samples to find the non-linear cumulative Abs energy curve from the lowest to highest in a temporal order. The significant changes in the gradient of this cumulative Abs energy curve are considered to be the moment of damage and failure region initiations.

#### Impact on cumulative Abs energy with the variation of mechanical load

**Fig 8:**
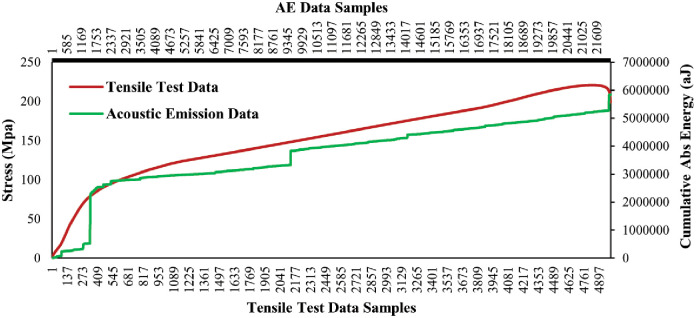
Average cumulative Abs energy versus average mechanical load of the 45° specimen.

**Fig 9:**
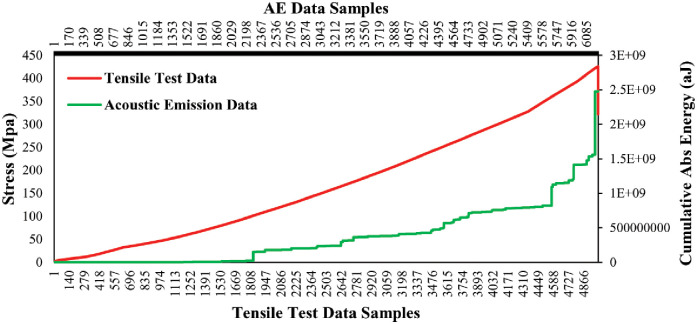
Average cumulative Abs energy versus an average mechanical load of the 90° specimen.

In this section, AE data samples were considered to observe the behavior of the average cumulative Abs energy curve over the changes of mechanical data samples. Both types of selected data samples is computed by averaging the data samples collected from the five tensile tests on a similar type of specimen. Figs 8 and 9 show the data correlation between AE data and mechanical data for two different orientations 45° and 90° of specimen respectively. The plots represent the initiations of the different phases in the gradients of the average cumulative Abs energy curves with the increment of the mechanical load for both lay-ups. Hence, it can be claimed that the AE data have a strong correlation with the mechanical data. Thus, we performed statistical analysis to validate and compute the correlation matrix between AE data and mechanical data for 45° and 90° specimens in the following section.

#### Correlation analysis between AE data and mechanical data based on statistical test

**Fig 10:**
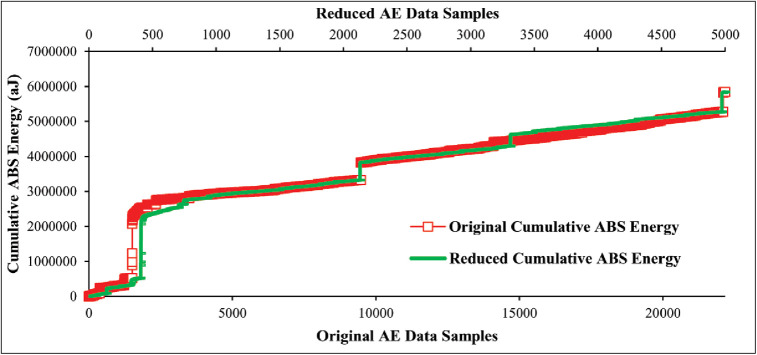
Correlation matrix between AE data and mechanical data collected from 45° specimen.

**Fig 11:**
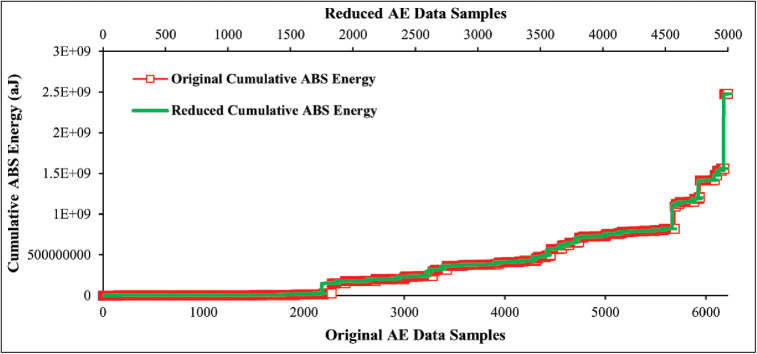
Correlation matrix between AE data and mechanical data collected from 90° specimen.

Correlation analysis is widely utilized as a statistical procedure to measure and assess the statistical relationship between two different continuous variables. Pearson’s correlation test was used to measure the association or relationship between the AE Data and mechanical data because it is based on the covariance method. Figs 10 and 11 present the correlation matrices between AE data and tensile test mechanical data for 45° and 90° specimens respectively. Both correlation matrices highlight the linear relationship of the Pearson’s correlation coefficients and histograms of the AE data and mechanical data. The results show that there is a strong positive linear relationship exist between them and Pearson’s correlation coefficients *R* = 0.98 for 45° specimen and *R* = 0.94 for 90° specimen. It is concluded that the two sets of data of both orientations were significantly correlated. Moreover, the obtained results meant that AE data can be used to characterize damage and failure regions that were characterized by the mechanical data to a reasonable extent. In order to classify the damage and failure of carbon fiber composite laminates, the proposed unsupervised partitioning clustering technique will be utilized on AE data with respect to the mechanical data in this paper. For that, the number of data samples should be equal for both AE data and mechanical data. However, the sampling rate of AE data was much higher than the mechanical data during our tensile tests. The reason is that the AE data are very sensitive to noise and acquire high-resolution AE features. Hence, an adaptive sampling technique should be applied to AE data to reduce the number of unnecessary samples before the proposed data clustering technique is performed.

#### Redundant AE data reduction based on adaptive sampling technique

**Fig 12:**
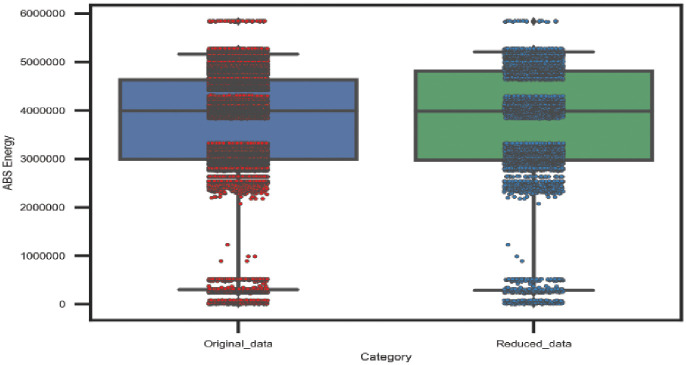
Performance comparison of original AE data and reduced AE data of 45° specimen using the proposed adaptive sampling technique.

**Fig 13:**
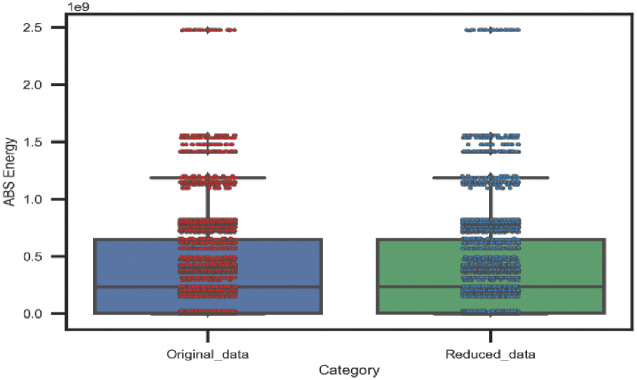
Performance comparison of original AE data and reduced AE data of 90° specimen using the proposed adaptive sampling technique.

In this section, we utilize our proposed adaptive sampling technique on AE data to eliminate a number of redundant data samples. The total number of collected original data samples is 22000 in the case of 45° specimen and 6200 in the case of 90° specimen presented in Figs 8 and 9. Afterward, the proposed technique is applied to original data samples of both lay-ups specimens and reduced to 5000 data samples that are equal to the collected mechanical data samples. Figs 12 and 13 show the originality between the original data and reduced data pattern after the unnecessary redundant data reduction using our proposed technique. The results show that the originality of the reduced AE data samples of both lay-ups specimens is almost equal to the original data samples because the deviation between the original and reduced data samples is less. Hence, based on the obtained results, it can be claimed that the original AE data samples have a strong temporal correlation, and thus our proposed technique performed well in terms of accuracy even after a significant amount of data reduction.

#### Statistical data analysis after unnecessary AE data reduction

**Fig 14:**
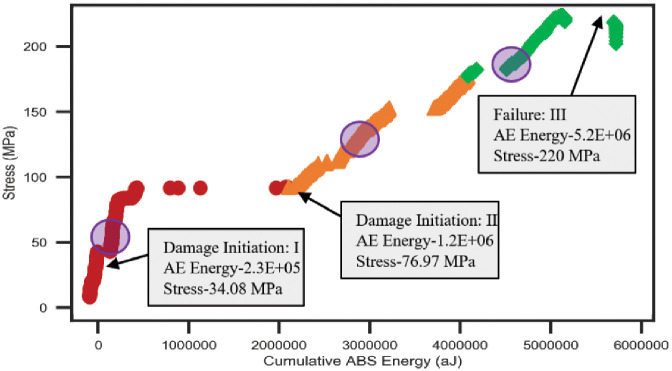
A comparison between original data and reduced data of 45° specimen by the adaptive sampling technique versus cumulative Abs energy.

**Fig 15:**
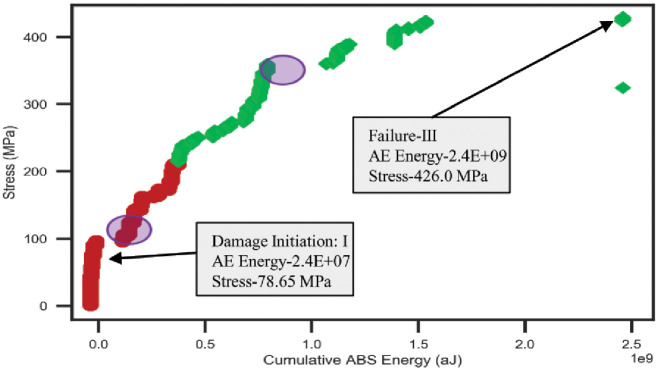
A comparison between original data and reduced data of 90° specimen by the adaptive sampling technique versus cumulative Abs energy.

The statistical properties of the original and reduced AE data are illustrated in this section to evaluate our proposed adaptive sampling technique. The original data samples (1*x*22000) and (1*x*6200) for the 45° and 90° specimens respectively as well as the reduced data samples (1*x*5000) for both lay-ups are considered. Figs 14 and 15 show the statistical properties of the distribution of AE data and the spread of data over the boxplots for the 45° and 90° specimens correspondingly. From the boxplots, it can be observed that the minimum, maximum, upper quartile, lower quartile, and median values of the distribution maintained almost the same as the original AE datasets for both lay-ups. Thus, it can be claimed that our proposed technique only eliminates redundant data by maintaining almost similar statistical properties.

#### Damage and failure characterization based on unsupervised clustering on AE data with respect to the mechanical data

**Fig 16:**
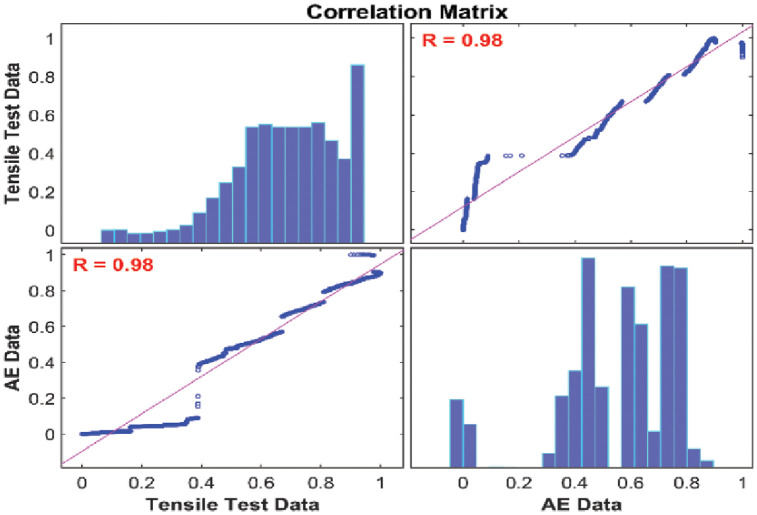
Classification of different regions for 45° specimen based on bivariate data clustering.

**Fig 17:**
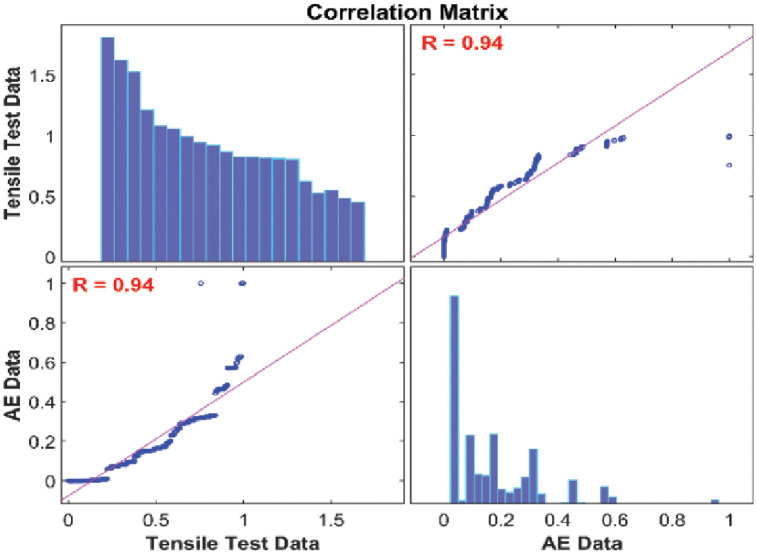
Classification of different regions for 90° specimen based on bivariate data clustering.

In this section, the proposed unsupervised clustering technique has been utilized on bivariate data to form the desired number of clusters for the damage and failure classifications of carbon fiber composite laminates. In bivariate data clustering, 5000 AE data samples (cumulative Abs energy) of each orientation are considered as a dependent variable and 5000 mechanical data samples (Stress) are selected as an independent variable. The desired number of clusters 3 and 2 are defined for the data samples of 45° and 90° specimens respectively. Afterward, the performance of our proposed clustering technique was investigated based on bivariate data analysis. Fig 16 represents the different groups of AE cumulative Abs energy with respect to the mechanical stress data of 45° specimen. The plot shows a line curve of cumulative Abs energy with three “red circles” that are represented the central points of the three different clusters. A set of AE data of each cluster characterizes a particular phase of degradation of carbon fiber composite laminates which had already been identified earlier based on mechanical data. The values of AE data and mechanical data for initiative damage and failure classification in three different regions are highlighted in the rectangular boxes in the plot. On the other hand, Fig 17 shows two different groups of AE data with respect to the mechanical data of 90° specimen. These two groups of data represent the two different regions named Region-I and Region-III defined based on the mechanical data. In this orientation, Region-II is unable to classify due to the strong linear behavior of the mechanical data over time. The parameters values of both regions are stated in the boxes of the graph. Hence, it can be claimed that the cumulative Abs energy is an important feature of AE data to classify the damage initiation and failure regions. Moreover, the proposed unsupervised clustering performs well in detecting damage initiation and failure regions of carbon fiber composite based on AE data and these regions are almost similar regions identified by the stress-strain curve.

#### Damage and failure characterization by the combination of AE data and mechanical data

In this section, the sentry function *f*(*x*) introduced by [21], has been utilized to validate our proposed clustering technique and increase the sensitivity of the damage and failure regions classification. The sentry function is defined as the logarithm of mechanical energy or stress *E*_*m*_(*X*_*i*_) to the AE energy or cumulative Abs energy $E_{AE}\left(X_{i}^{{}^{\prime }}\right)$. The sentry function characterizes the damage and failure regions based on the combination of mechanical energy and AE energy. Based on the state of the damage in the composite material structure, the sentry function shows one of the four following trends:

1. Increasing trend: it shows that the structure is still intact and no damage or some micro damages occurred in the material.
2. Sharp drop: it reveals that huge damage occurred in the material.
3. Constant trend: It demonstrates that there is a balance between the degrading mechanisms, like damages, and the strengthening mechanisms, like fiber bridging.
4. Gradually decreasing: It is emphasizing that the load-carrying capability of the composite structure is losing gradually.

**Fig 18:**
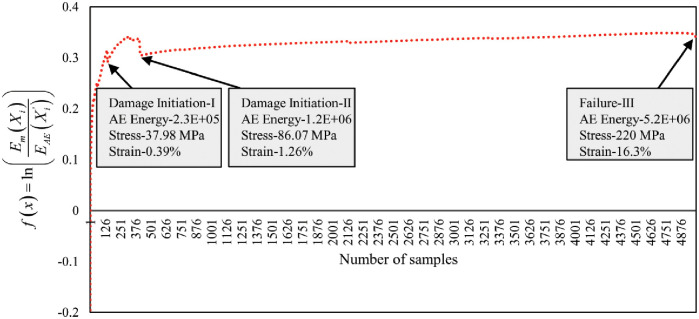
Sentry function analysis by the combination of AE and mechanical data of 45° specimen.

**Fig 19:**
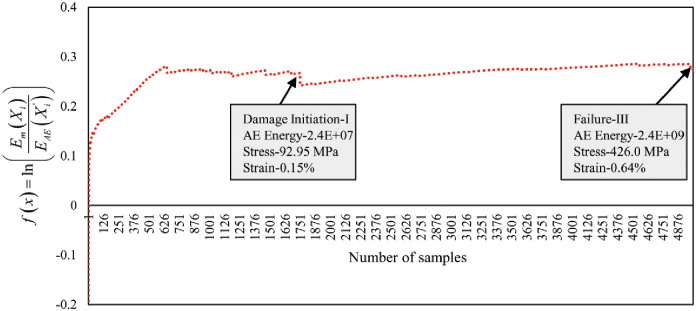
Sentry function analysis by the combination of AE and mechanical data of 90° specimen.

Accordingly, the first big drop in the sentry function curve is considered as the moment of the damage initiation. Figs 18 and 19 show the curves of the sentry function of 45° and 90° specimens. The increasing graph trend up to damage initiation-I indicates the elastic region (Region I) where the fiber reinforcement was still bonded strongly to the matrix with the ability to return to the original shape when the load is released. The curve line within the damage Initiation-I and damage Initiation-II shows two events that happened in the specimen structural bonding such as matrix micro damaging and matrix de-bonding from the fiber respectively. Afterward, the specimen reaches the maximum yield strength. Then the trend-line shows almost constant trends until the Failure-III event has occurred. During this event, the specimen underwent a strain hardening phase (Region III) where the fiber reinforcement was stretched gradually to the load until reaching the UTS and severe fiber pull-out occurred and broke. It can be observed that the above-mentioned trends exist in the following curves in Figs 18 and 19 of both lay-ups which characterize the different state of the composite material structure.

#### Damage and failure characterization based on AE data

**Fig 20:**
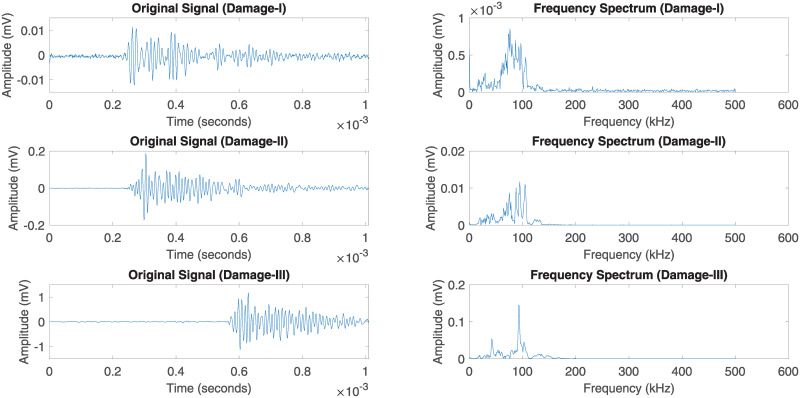
Original AE signals for several damage regions of the 45° specimen and their corresponding frequency spectrum.

**Fig 21:**
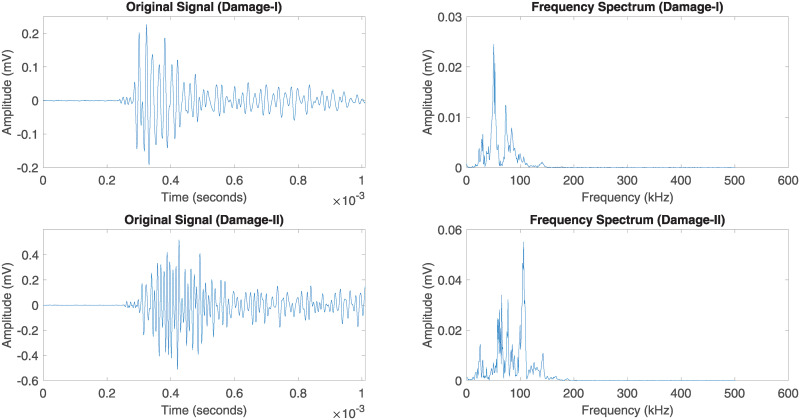
Original AE signals for several damage regions of the 90° specimen and their corresponding frequency spectrum.

**Fig 22:**
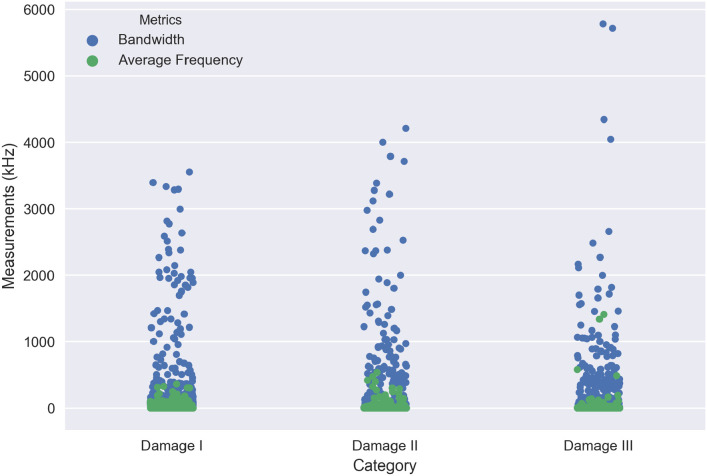
A comparison between bandwidth and average frequency for three different regions of the damage in 45° lay-up specimen.

**Fig 23:**
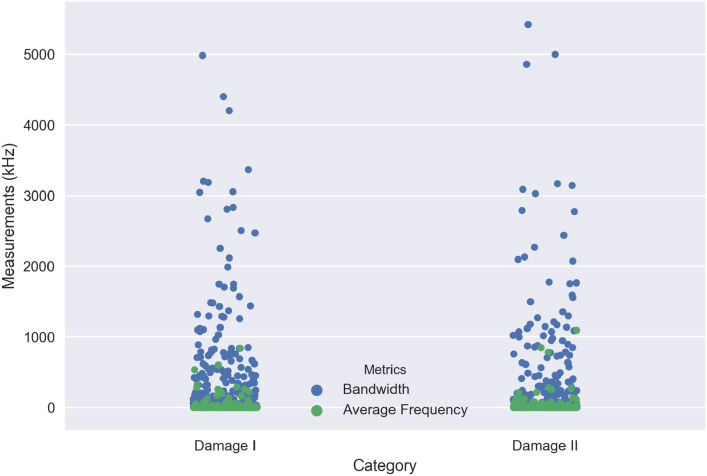
A comparison between bandwidth and average frequency for two different regions of the damage in 90° lay-up specimen.

In this section, an individual AE waveform has been selected for each damage region and interpreted the particular waveform based on time-frequency analysis. It allows the analytical extraction of temporal and spectral information from a complex group of AE data. Each damage initiation of the response AE signal *x*(*t*) is transformed based on Fast Fourier Transform (FFT) to extract the frequency spectrum. Figs 20 and 21 illustrate the original AE signals and energy spectrum of different damage and failure regions for carbon fiber specimens in 45° and 90° lay-ups. The damage and failure classifications of can be achieved by means of the energy (*E*), root-mean-square (RMS) Bandwidth (*B*) and the average frequency (*ω*_*c*_). These parameters are explained below:

$$\begin{array}{c} E=\frac{1}{2\pi }\sum_{n=0}^{N-1}{\left|X_{n}\right|}^{2}, \end{array}$$ (4)

$$\begin{array}{c} \omega_{c}=\frac{\sum_{n=0}^{N-1}\omega_{n}{\left|X_{n}\right|}^{2}}{2\pi E}, \end{array}$$ (5)

$$\begin{array}{c} B=\sqrt{\frac{1}{E}\sum_{n=0}^{N-1}{\left(\omega_{n}-\omega_{c}\right)}^{2}{\left|X_{n}\right|}^{2}}, \end{array}$$ (6)

where *X*_*n*_ and *ω*_*n*_ are the FFT and angular frequency of the response signal *x*(*t*) in discreet time *y*(*n*) respectively and *N* is the number of discrete time-points. Fig 22 shows the comparison between bandwidth and average frequency for three different regions of the damage in 45° lay-up specimen. It can be observed that the AE activity varies in different stages of damage. As observed in Fig 22, the bandwidth ranged from 1.36kHz to 3553.72kHz for “Damage I”, 0.83kHz to 4212.61kHz for “Damage II” and 0.14kHz to 5785.73kHz for “Damage III” as well as the average bandwidth is 312.23kHz, 248.09kHz and 216.43kHz for “Damage I”, “Damage II” and “Damage III” respectively. In contrast, the various ranges of average frequency can be seen which are from 0kHz to 359.9561309kHz, 0kHz to 537.56kHz, 0kHz to 1409.01kHz for “Damage I”, “Damage II” and “Damage III” accordingly. On the other hand, Fig 23 presents the comparison between bandwidth and average frequency for two different regions of the damage in 90° lay-up specimen. According to the obtained measurements in Fig 23, the bandwidth ranges can be observed from 0.14kHz to 4981.69kHz for “Damage I” and 0.25kHz to 5422.55kHz for “Damage II” as well as the average bandwidth is 240.56kHz and 209.05kHz for “Damage I” and “Damage II” respectively. Apart from it, two different ranges of average frequency are computed which are from 0kHz to 836.56kHz and 0kHz to 1090.49kHz for “Damage I” and “Damage II” correspondingly. According to the obtained results, the different levels of damage can be classified utilizing the different ranges of bandwidth and average frequency. It can also be observed that the activities of AE parameters increase in a different regions of damage initiations accordingly.

## Concluding remarks

Our findings and analysis confirm the fact that data clustering is very useful for detecting early-stage damage and failure modes of CFRP specimens. The proposed bivariate data clustering contributes to a better understanding of the structural behavior of CFRP specimens with high sensitivity in damage monitoring, hence making it more useful for non-destructive structure health monitoring applications.

The several modes of damages monitor using the AE data recorded by a piezoelectric sensor attached to CFRP specimens with different lay-up patterns, whilst undergoing tensile tests. The mechanical information of the CFRP specimen combines with AE data to monitor the initiation and progress of the mode of damages in the tested specimens. According to the mechanical stress-strain curve, the behavior of the 45° specimen falls under the label of ductile properties, while the 90° specimen is prone to brittle behavior. The validation of the mechanical data-based definitions was rectified using SEM analysis. The proposed clustering technique characterizes the mode of damages of CFRP specimens into different clusters with respect to the mechanical data. The validation of cluster-based classification is performed by the sentry function analysis with the increment of the sensitivity of the several states of damage initiations. The main contribution of this paper is to prove that there is a strong correlation between AE and stress-strain data in a specific lay-up but no correlation in different lay-up for the same carbon fiber composite materials. Characterizing the several acoustic responses of the same carbon fiber material under various stress states using our bivariate data clustering technique which can be utilized for SHM in real composite structures. Moreover, the time-frequency spectrum and sentry function analysis provide the acoustic responses and dissimilar AE features of different modes of damage initiation regions that can be used to develop the early-stage damage detection and failure prediction tool for the composite materials. Therefore, it can be concluded that the introduced methods in this paper are successful in the characterization process to enhance the classification of the damage mechanisms in actual occurring modes of delamination. The extension of this work will focus on the utilization of other available important AE features to classify the modes of damage of different composite materials.
